# The Splenic Marginal Zone in Children Is Characterized by a Subpopulation of CD27-Negative, Lowly IGHV-Mutated B Cells

**DOI:** 10.3389/fimmu.2022.825619

**Published:** 2022-01-27

**Authors:** Artur Kibler, Bettina Budeus, Ralf Küppers, Marc Seifert

**Affiliations:** Institute of Cell Biology (Cancer Research), University of Duisburg-Essen, Essen, Germany

**Keywords:** human, B cell, marginal zone (MZ) B cell, aging, spleen

## Abstract

Young children and older adults suffer from enhanced susceptibility to infections with blood-borne pathogens. An essential step towards immunity is the establishment of a splenic marginal zone (sMZ), which is immature at below 2 years of age. At approximately 5 years of age, an adult level of protection is reached but wanes again in older adults. Although the infant sMZ is thought to contain mostly naïve B cells, memory B cells are recruited to and recirculate from the sMZ throughout life, and class-switched sMZ B cells dominate in older adults. For a better resolution of naïve versus memory B-cell subset accumulation in the sMZ, we performed a single cell-based gene expression analysis of (CD21^high^IgM^high^) sMZ B cells among five healthy donors (age 3 to 48 years) and validated the sMZ B-cell subset composition by flow cytometry of 147 spleen biopsies (age 0 to 82 years). We identified a major sMZ B-cell subpopulation, which is abundant at birth but decreases with age. These cells lack CD27 expression but carry a weak-to-intermediate memory B-cell signature. These CD27^neg^ sMZ B cells are either IGHV-unmutated or carry only a few IGHV mutations early in life but show average memory B-cell IGHV mutation frequencies (>3%) in adults. The activation and proliferation potential of CD27^neg^ sMZ B cells is significantly above that of non-sMZ B cells already in children. Our study suggests that the human sMZ B-cell pool changes with age, encompassing a major population of lowly Ig-mutated CD27neg but antigen-experienced B cells early in life.

## Introduction

Human splenic marginal zone (sMZ) B cells are located at the interface of the red and white pulp. This specialized microenvironment maintains a “state of active readiness” among sMZ B cells (1). sMZ B cells require NOTCH2 signaling for their development (2, 3) and are important in the defense against blood-borne pathogens (4). It is not clarified why infants and children below 5 years of age and older adults (>60 years) suffer more frequently from infections with blood-borne pathogens, nor the characteristics of a protective sMZ B-cell compartment between these extremes of age (5).

The human spleen is considered immunologically immature at below 2 years (6, 7); although sMZ B cells are principally detectable (8), they have been described as antigen naïve B cells (NBCs) based on the lack of CD27 expression (7). Previous studies on human sMZ B-cell development suggest that unique diversification mechanisms (9, 10) and distinct sMZ B-cell precursors (3) exist in infants. Our own data support that infant B-cell subsets differ substantially in their gene expression profiles from adult B cells: human ontogenetically early B cells provide accelerated but transient immune responses and are IGHV gene-unmutated (11). Throughout life, the accumulation, homeostatic expansion, and recirculation of IGHV gene-mutated B cells shape the sMZ B-cell pool (8). Notably, homing of memory B cells (MBCs) to the sMZ enhances at approximately 2 years and reaches an adult level at approximately 5 years of age, which coincides with efficient protection from infections with encapsulated bacteria (5, 12). In older individuals, the aged immune system is compromised on multiple levels (5), including a significantly reduced IGHV gene repertoire, where large B-cell clones outcompete the smaller ones (8). We assume that a protective sMZ compartment is affected by age-related changes in the composition of the sMZ B-cell pool, as indicated by, e.g., the abundance of large, class-switched MBC clones in old age (8).

A major protective function of the sMZ is associated with IgM-expressing B cells, mostly IGHV-mutated and antigen-experienced in adults, and their associated IgM-antibody titers (4). To assess the heterogeneity of IgM-expressing B-cell subsets in a protective sMZ, and whether this may change with age, we investigated CD21^high^IgM^high^ sMZ B cells from five healthy donors aged 3, 5, 12, 27, and 48 years by multiplex single-cell qRT-PCR, focusing on known genes best differentiating NBCs, IgM-MBCs and IgG-MBCs, and bulk sMZ B cells. As others and we described a very low abundance of IGHV-mutated IgM^+^ B cells in donors below 2 years and class-switched MBCs dominating at above 60 years, very young and old spleens were disregarded in the 96 cell-based expression profiling. Throughout age, each analyzed sMZ B cell carries a NOTCH-dependent signature, and all sMZ B cells investigated carried a variable signature of antigen-experienced IgM MBCs. According to our analysis, the IgM^+^ sMZ B-cell compartment is composed of two major B-cell subsets, which include typical IgM MBCs, but also a subpopulation of CD27^neg^ B cells with lowly mutated IGHV genes and a weak-to-intermediate gene expression signature of antigen-experienced B cells. A decrease of this CD27^neg^ subset and an increase in NOTCH signaling best explained the influence of aging from young children to adults in our data set.

## Materials and Methods

### Human Samples

Splenic biopsies (**Table S1**) were collected from leftover material from the Institute for Transfusion Medicine at the Medical School Essen, as approved by the ethics committee of the Medical Faculty of the University of Duisburg-Essen, Germany. All individuals died of non-infectious and non-malignant causes, i.e., anoxic brain damage, cardiac arrest, cerebral trauma, bleeding, or infarction. Acutely and chronically infected (such as HIV, HBV, and HCV) individuals were excluded.

### Mononuclear Cell Isolation, Magnetic-Activated Cell Sorting, Fluorescence-Activated Cell Sorting, and Cell Sorting

Mononuclear cells were isolated by Ficoll density gradient centrifugation and CD19^−^ magnetic-activated cell sorting (MACS) (Miltenyi Biotec). B cells were incubated with anti-IgD-PerCP-Cy5.5 (clone IA6-2), anti-IgM-FITC (G20-127), anti-CD20-BV421 (2H7), anti-CD21-PE (B-ly4), anti-CD23-APC (M-L233), and anti-CD27-PE-Cy7 (all BD Biosciences, O323) antibodies. B cells were analyzed on a CytoFLEX S flow cytometer (Beckman Coulter) using the CytExpert software V2.4 (Beckman Coulter). Sort purification was performed on a FACSAria III or FACSAria Fusion cell sorter (BD Biosciences) equipped with BD FACSDiva software (BD Biosciences). CD21^high^IgM^high^ (CD20^+^IgM^high^CD21^high^), IgM MBCs (CD20^+^IgM^+^CD27^+^CD23^−^CD21^+^), NBCs (CD20^+^IgM^+^CD27^neg^CD23^+^CD21^+^), CD27^neg^ sMZ (CD20^+^IgM^high^CD27^neg^CD21^high^), and CD27^+^ sMZ (CD20^+^IgM^high^CD27^+^CD21^high^) B cells were isolated. Purity of sorted B-cell subsets was >98%. In single-cell sorting experiments, every single cell was sorted into individual wells of a 96-well PCR plate, using the built-in protocol within the FACSDiva software (device, 96-well plate; precision, single-cell; nozzle, 100 µm).

### Single-Cell qRT-PCR

Single-cell gene-expression experiments were performed using 96.96 Dynamic Array (Fluidigm Inc.). Single cells were sorted into individual wells of 96-well PCR plates and preloaded with 5 µl/well of CellsDirect One-Step qRT-PCR mix (Thermo Fisher); then each well was supplemented with 1 µl of SuperScript-III RT/Platinum Taq (Invitrogen) and a mixture of 96 pooled TaqMan assays (Fluidigm Inc., 5 µM each). Single-cell mRNA was reverse transcribed (50°C for 15 min, 95°C for 2 min) and pre-amplified for 22 cycles (95°C for 15 s, 60°C for 4 min). Libraries and probes were transferred into 96.96 Dynamic Array integrated fluidic circuits (IFCs) for qRT-PCR on a BioMark HD instrument (both Fluidigm Inc.) following the manufacturer’s instructions.

### Sanger Sequencing of Cloned IGHV Genes

Human CD27^+^ and CD27^neg^ sMZ B cells were isolated in aliquots of 10^4^ cells each from four donors. RNA was extracted (RNeasy micro kit, Qiagen); mRNA was reverse transcribed using random hexamer primers with the Superscript-III-RT (Invitrogen) according to the manufacturer’s protocol. Rearranged IGHV genes were amplified using a semi-nested PCR protocol with custom IGHV1-6 primers (FR1) and IGHJ primers, as described previously (11). PCR products were purified, cloned into a pGEM-T easy vector (Promega), and transformed into JM109 bacteria, which were cultivated in the presence of ampicillin, X-Gal, and IPTG. Per population and donor, 100 white colonies were randomly picked, plasmid DNA was isolated, and IGHV sequences were retrieved using Sanger sequencing. Identification of the IGHV gene was performed in R (www.R-project.org/) using rBLAST and the ImMunoGeneTics (IMGT) database (www.imgt.org). Mutation frequencies were calculated based on the nucleotide exchanges in the IGHV region in comparison with the most similar allelic germline variant present in IMGT.

### *In Vitro* Cell Culture and Functional Analysis

B cells were cultured in Roswell Park Memorial Institute (RPMI) 1640 medium supplemented with 20% fetal bovine serum (Pan Biotech), 100 U/ml of penicillin, and 100 µg/ml of streptomycin at 37°C and 5% CO_2_. B cells were left unstimulated or activated by T cell-dependent (TD) stimulation using 0.03 µg/µl of anti-Ig (Jackson ImmunoResearch) and 1 µg/ml of CD40 ligand hemagglutinin with 5 ng/ml of anti-hemagglutinin antibodies (R&D Systems). T cell-independent (TI-I) stimulation was mimicked by incubation with 2.5 µM of cytosine-phosphate-guanine oligonucleotide type B (InvivoGen). Phenotypic analyses were performed directly upon B-cell purification and after 24 h of incubation in the presence of TI-I or TD stimulation. In proliferation assays, cells were pulsed with eFluor670 (Invitrogen) at a concentration of 5 µM, stimulated with TD or TI-I stimulation, and analyzed after 48, 72, and 96 h of incubation.

## Results

**Figure 1:**
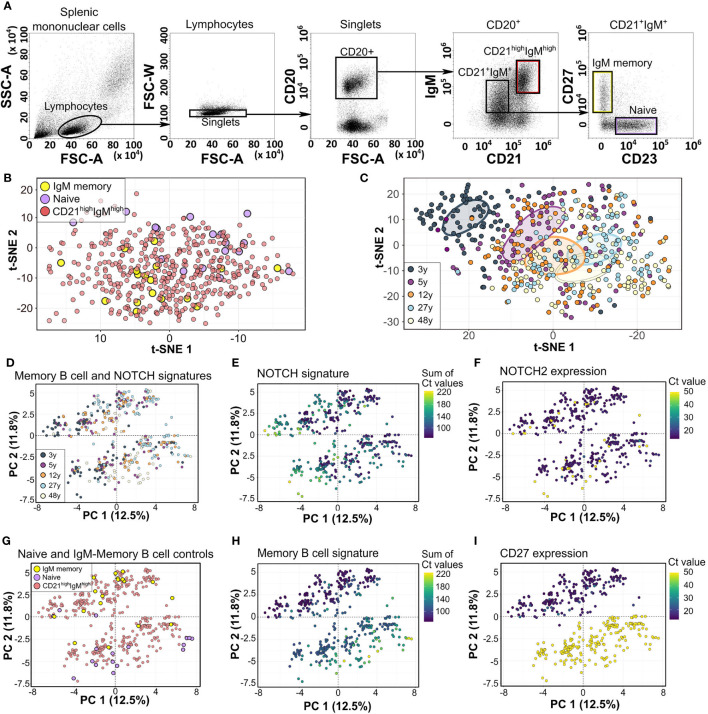
Fluidigm 96.96 analysis of human splenic CD21^high^IgM^high^ zone B cells. **(A)** Gating strategy of human splenic NBCs, IgM MBCs, and CD21^high^IgM^high^ B cells. **(B)** Unsupervised t-SNE analysis of 95 genes and 460 cells from 5 donors. **(C)** Unsupervised t-SNE analysis of most differentially expressed genes (MANOVA q < 0.05, 31 genes, **Table S2**) among sMZ B cells from five donors. Colored circles denote the centers of mass (top 10%) as determined by ggplot. Individual donors are shown in **Figure S1A**. **(D)** Supervised PCA of 23 genes associated with hallmark molecules distinguishing NBCs from MBCs, sMZ B cells, and NOTCH signaling genes (**Table S2**). Individual donors are shown in **Figure S1B**. **(E)** Expression of a NOTCH signature (sum of Ct values, **Table S2**). **(F)** Expression of NOTCH2 (Ct value). **(G)** Location of naïve and MBC control cells. **(H)** Expression of an MBC signature (sum of Ct values). **(I)** Expression of CD27 (Ct value), in the PCA shown in panel **(D)** NBCs, naïve B cells; MBCs, memory B cells; t-SNE, t-distributed stochastic neighbor embedding; MANOVA, multivariate ANOVA; PCA, principal component analysis.

Single-cell analysis is suitable to investigate the cellular composition of immune cell populations (13). Here, we performed Fluidigm 96.96 Dynamic Array to investigate the composition of the IgM-expressing sMZ compartment of five donors (3 to 48 years). With the use of single-cell deposition, 88 sMZ of B cells (CD21^high^IgM^high^), four splenic non-MZ IgM-MBCs (CD21^+^IgM^+^CD23^−^CD27^+^), and four splenic NBCs (CD21^+^IgM^+^CD23^+^CD27^−^) were isolated from each donor (**Figure 1A**) and subjected to multiplexed single-cell qRT-PCR. This approach evaluates the expression of up to 96 genes in 96 cells per donor. It is thus limited to the quantification of sufficiently abundant B-cell subsets and only preselected biological processes. We therefore focused on genes previously shown to distinguish IgM MBCs and NBCs (e.g., ABCB1, CD27, and FCER2), markers for sMZ B cells (e.g., CR1, CR2, and CD1C), and transitional B cells (CD5, CD24, and CD38) (3, 9, 11, 14, 15). Moreover, we selected genes associated with Ig diversification and the germinal center reaction (e.g., AICDA and BCL6), NOTCH signaling (e.g., HES1, DTX1, and PSEN1), and microenvironmental interactions (e.g., ITGB2, CCR6, and CXCR5). Our selection preferred cell surface molecules to allow for flow cytometric validation of putative subsets (**Table S2**). Quality control of Fluidigm 96.96 data excluded empty wells and doublet cells (double transcript density), or genes with Ct < 5, or limit of detection (LOD) <24. AICDA expression was undetectable in all cells.

Unsupervised analysis of the whole data set (95 genes from 460 cells of five spleens, as 1 gene and 20 cells were removed during quality control) by dimensionality reduction and projection [t-distributed stochastic neighbor embedding (t-SNE) or principal component analysis (PCA)] separated NBCs from IgM MBC controls but did not identify distinct clusters among sMZ B cells (**Figure 1B**). We performed an unsupervised selection of the most differentially expressed transcripts among all sMZ B cells from all donors [3 to 48 years, multivariate ANOVA (MANOVA)] resulting in 31 transcripts (**Table S2**). The t-SNE clustering of this selection separated the youngest and oldest donor best, and the remaining donors (5, 12, and 27 years) were integrated in an age-dependent manner in this young-to-old map (**Figure 1C**). This clustering suggested both an age-dependent shift of transcription profiles (see the 3-, and 5- to 12-year-old distribution; **Figure 1C** and **Figure S1A**) and an age-dependent change in cellular composition (each donor is composed of variable amounts of “child” and “adult” B cells, **Figure 1C** and **Figure S1A**). The 31 genes underlying this map included CCR6, CD80, FCER2, IGHD, CD5, TCL1A, PSEN1, NOTCH1, and DTX1. Next, we investigated the impact of NBC, MBC, or NOTCH signatures on sMZ subset composition in a supervised manner (23 genes, **Table S2**) repeatedly identifying a major impact of age in the PCA (PC1; **Figures 1D**, **S1B**). The major contributing genes to PC1 included BACH2 and RFTN1 and NOTCH signaling genes (PSEN1, DTX1, and HES1), which were gradually expressed to be higher among older donors (12, 27, and 48 years; **Figure 1E**); only NOTCH2 expression was independent of age (**Figure 1F**). PC2 separated two distinct clusters among each donor (**Figure 1D**) by the expression of CD27, CD24, PTPRJ, NT5E, and IGHD. These genes distinguish IgM MBCs from NBCs in human peripheral blood (PB) (16) and showed the highest expression among the sMZ B-cell cluster co-localizing with IgM MBC control cells (**Figures 1G, H**). The remaining sMZ B cells, which all lacked CD27 expression (**Figure 1I**), also carried this IgM MBC signature, albeit at reduced and more variable intensity (**Figure 1H**). In addition, although the CD27^neg^ sMZ B-cell cluster co-localized with NBC controls, the respective expression profiles were dominated by IgM MBC-related genes (CCR6, CD44, PTPRJ, etc.; see **Table S2**). Only 15/420 sMZ B cells carried an NBC signature, as defined by the most differentially expressed genes between IgM MBC and NBC controls (ABCB1, CD200, and FCER2). We conclude that the human sMZ B-cell compartment between 3 and 48 years of age includes a population of CD27^neg^ B cells, which barely show transcriptional similarity to antigen-unexperienced B cells.

**Figure 2:**
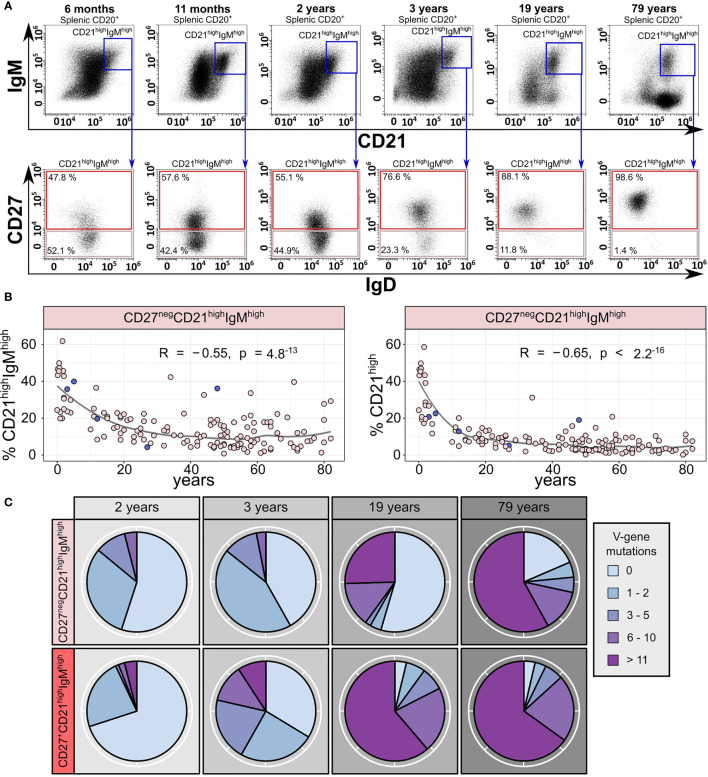
Two subsets of human splenic CD21^high^IgM^high^ zone B cells exist, and their abundance changes with age. **(A)** Representative gating strategy of CD27^neg^CD21^high^IgM^high^ and CD27^+^CD21^high^IgM^high^ sMZ B cells from six donors (6 months, 11 months, 2 years, 3 years, 19 years, and 79 years). **(B)** Age-related frequency of splenic CD27^neg^CD21^high^IgM^high^ B cells among IgM-expressing (left panel) and total sMZ B cells (right panel; n = 147; 104 donors originally published in Kibler et al. (8); 43 additional donors). The circles corresponding to the five donors used for Fluidigm 96.96 analysis are marked in dark blue. Statistics denote Pearson’s correlation coefficient. **(C)** Number of IGHV gene mutations per IGHV sequence in CD27^neg^CD21^high^IgM^high^ and CD27^+^CD21^high^IgM^high^ B cells from four donors (2 years, 3 years, 19 years, and 79 years).

We aimed to validate the presence of a CD27^neg^ sMZ B-cell population and determined their age-related frequency by flow cytometry of human splenic B cells from 147 donors (**Figures 2A, B**; 104 originally published in Kibler et al. (8) and 43 additional donors). This analysis confirmed the presence of two subsets among CD21^high^IgM^high^ sMZ B cells, one IgD^low^CD27^+^ and the other IgD^high^CD27^neg^, with the latter representing up to 50% of sMZ B cells in young children but decreasing with age (**Figures 2A, B**). Class-switched sMZ B cells continuously accumulate in donors >2 years (8) (**Figure 2A**, top panel: IgM^neg^CD21^high^ B cells), which also contributes to the relative decline of CD27^neg^ sMZ B cells within the CD21^high^ sMZ compartment (**Figure 2B**, bottom). Note that the 48-year-old donor, which was analyzed by Fluidigm 96.96 arrays, contained an unusually high frequency of CD27^neg^ sMZ B cells (**Figure 2B**).

We aimed to characterize the two major sMZ B-cell subsets on a molecular level. To this end, we collected up to 90 productive IGHV gene rearrangements from 10^4^ purified CD27^neg^ and CD27^+^ sMZ B cells each, from 4 donors (2, 3, 19, and 79 years) by bulk PCR, cloning, and Sanger sequencing. In both children, the average IGHV gene mutation frequency of CD27^neg^ sMZ B cells was below 0.5%, but it was 2.2% and 3.9% for the B cells from the adults (**Figure 2C**). The average IGHV gene mutation frequency of CD27^+^ sMZ B cells was below 1% in the 2-year-old child, 1.5% in the 3-year-old child, and above 5% in the 19- and 79-year-old donors (**Figure 2C**). The IGHV gene mutation load among CD27^neg^ sMZ B cells varied substantially, similar to the variation of the IgM MBC signature in the CD27^neg^ cluster (**Figure 1F**). No clonal expansions were detectable (95% nucleotide homology of the CDR3 and identical IGHV gene). IGHV gene PCR and cloning error frequency were <0.2% in a cell line.

**Figure 3:**
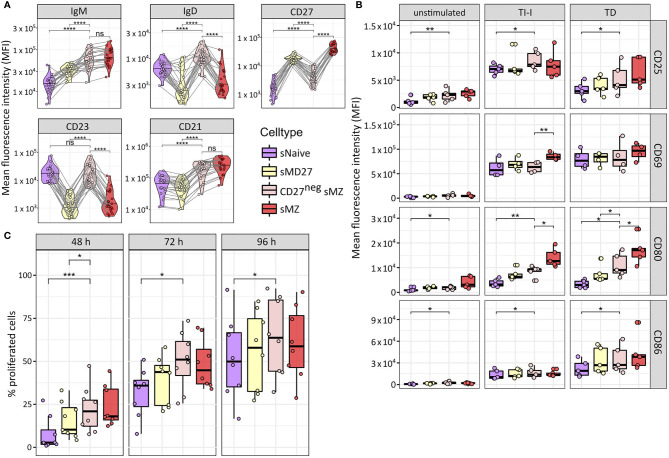
Phenotypic and functional characterization of splenic CD27^neg^CD21^high^IgM^high^ B cells. **(A)** Surface expression of IgM, IgD, CD21, CD23, and CD27 among splenic naïve, IgM memory, CD27^neg^CD21^high^IgM^high^, and sMZ B cells as determined by flow cytometry using the gating strategy as in **Figures 1A**, **2A**. B-cell subsets from the same donor are connected with lines. **(B)** Surface expression of CD25, CD69, CD80, and CD86 among resting B-cell subsets or upon 24 h of *in vitro* stimulation (TI-I and TD). Color code as in panel **(A, C)** Fraction of proliferated cells (eFluor670^low^) upon 48, 72, or 96 h of *in vitro* culture with TD and TI-I stimulation. Color code as in panel **(A)** Statistical significance was calculated by Wilcoxon rank-sum test using paired samples (*p ≤ 0.05; **p ≤ 0.005; ***p ≤ 0.001; ****p ≤ 0.0001; ns, p > 0.05).

We analyzed the surface expression of selected markers with differential expression among NBCs, IgM-MBCs, and the two sMZ B-cell subpopulations (**Figure 3A**), supporting the distinctness of CD27^neg^ sMZ B cells from NBCs and MBCs. No statistically significant differences were observed between infant- and adult-derived subsets. Upon *in vitro* stimulation, CD27^neg^ sMZ B cells quickly induced co-stimulatory molecules, such as CD80 and CD86, similar to CD27^+^ sMZ B cells (**Figure 3B**). Moreover, a large fraction of CD27^neg^ sMZ B cells proliferated already after 48 h of *in vitro* stimulation (**Figure 3C**). Even after 96 h of stimulation, CD27^neg^ sMZ B-cell proliferation capacity is more similar to IgM-MBCs and sMZ B cells than to NBCs (**Figure 3C**).

We conclude that CD27^neg^ sMZ B cells represent a distinct, young age-associated subset among human sMZ B cells, which differs from antigen-unexperienced B-cell subsets by phenotype, expression profile, and IGHV mutation load, and finally, shows a response potential that is most similar to that of the CD27^+^ sMZ B-cell subset.

## Discussion

Human sMZ B cells combine features of innate and adaptive immune cells (1, 17). These cells provide protection from life-threatening infections with encapsulated bacteria, thought to be mainly derived from the protective function of IgM (4). However, young children and older adults (>60 years) suffer more frequently from such infections despite the presence of sMZ B cells (5, 8). Thus, the protective potential of the human sMZ changes with age for yet unknown reasons. We previously showed that class-switched sMZ B cells accumulate over time and Ig repertoire diversity decreases (8). We assumed that the cellular composition might affect the IgM^+^ sMZ B-cell compartment. We addressed this hypothesis in a supervised manner by single-cell qRT-PCR. We focused on the contribution of well-defined gene sets associated with NBCs, MBCs, and sMZ B cells; microenvironmental interaction; and NOTCH signaling.

Our data suggest that B lymphocyte composition, NOTCH signaling intensity, and antigenic experience are influence the heterogeneity of the sMZ B-cell compartment in the age span from children to adults. Our data indicate that sMZ B-cell composition changes mostly during childhood and adolescence and is more stable in adults. The vast majority of human adult sMZ B cells express highly mutated IGHV genes and include IgM- and class-switched populations, both expressing the MBC marker CD27 (15, 16). Infant sMZ B cells, however, mostly lack class-switched B cells but include an abundant population of CD27^neg^CD23^+^IgM^high^IgD^high^ sMZ B cells. These CD27^neg^ sMZ B cells express a memory B-cell signature, albeit at medium intensity, and are largely IGHV-unmutated early in life but mostly include IGHV-mutated B cells in adults. In line with previous work (18), AICDA expression was undetectable in all sMZ B cells analyzed, supporting that IGHV mutations are acquired outside of the sMZ microenvironment. Moreover, the IgD^high^CD23^+^ phenotype of the CD27^neg^ sMZ B-cell subset may be related to more recent or less effective antigen stimulation compared with conventional sMZ B cells. IgM is upregulated and IgD is downregulated upon B-cell activation (19), which is in line with the Ig-mutation frequency and expression of IgD and CD23 by CD27^neg^ sMZ B cells.

Transcriptome analysis of bulk isolated NBCs, MBCs, and sMZ B-cell subsets identify thousands of differentially expressed genes in pairwise comparisons (14, 15). The current analysis of only 96 transcripts does not allow in-depth characterization of sMZ B-cell subsets, both immunologically and technically: note that 4/20 IgM MBC controls and 3/20 NBC controls located at the CD27^neg^ and CD27^+^ clusters, respectively, are counterintuitive to their respective isolation strategy. Thus, our approach is unsuitable to deduce a potential contribution of germinal center-dependent, extrafollicular, or primary diversifying immune mechanisms to the observed sMZ B-cell heterogeneity.

A significant fraction of IGHV-unmutated B cells with IgD^high^CD27^neg^ phenotype is detectable in infants but wanes with age. Note that an IGHV-unmutated B cell does not rule out mutations in Ig light chains or in the CDR3s, which were not assessed in the study. However, as IGHV-mutated sMZ B cells are detectable as soon as 2 weeks after birth (youngest donor in the cohort), and NOTCH2-dependent recruitment to the sMZ possibly favors antigen-experienced B cells (from intra- or extrafollicular responses) over NBCs, we speculate that antigen-unexperienced NBCs are rare, possibly only in infant B cells recruited to the sMZ (**Figures 2A, B**). Lowly Ig-mutated CD27^neg^ memory B cells were previously reported to be frequent in the PB of infants and children (20–22). We assume that these early memory B cells, which potentially derive from immature immune responses in young children, may be recruited into the sMZ and, thus, represent a source for the frequent CD27^neg^ sMZ B cells in the spleen of children. In line with this, our analysis suggests that virtually the entire sMZ B-cell subpopulation (including putative NBCs) shows a signature of antigen-experienced B cells, as defined by IgM-MBC controls. This signature may be imposed from the sMZ microenvironment, but likely also through a low level of stimulation by various antigens (T cell independent, T cell dependent, foreign, or self) that pass through this histologically exposed structure. Our results support previous suggestions that the human sMZ harbors multiple non-naïve B-cell subsets (23).

In summary, our Fluidigm 96.96 analysis suggests an age-dependent change in the cellular composition of sMZ B cells, starting already in young children. Moreover, we detected differentially expressed molecular signatures of MBCs and NOTCH signaling among single sMZ B cells and identified a corresponding subpopulation of CD27^neg^ sMZ B cells, which is abundant early in life but decreases with age. In young children, this CD27^neg^ sMZ B-cell population includes a large fraction of lowly IGHV gene-mutated cells and a marginal signature of MBCs, indicating previous antigen encounter.

## Data Availability Statement

The raw data supporting the conclusions of this article will be made available by the authors, without undue reservation.

## Ethics Statement

The studies involving human participants were reviewed and approved by the ethics committee of the Medical Faculty of the University of Duisburg-Essen, Germany. Written informed consent to participate in this study was provided by the participants’ legal guardian/next of kin.

## Author Contributions

AK designed and carried out most of the experiments and prepared the manuscript. BB developed and performed bioinformatics and statistical analyses. RK, BB, and MS developed the concept, designed experimental strategies, helped with data evaluation, and prepared the manuscript. All authors contributed to the article and approved the submitted version.

## Funding

This work was supported by the Deutsche Forschungs-gemeinschaft through grants SE1885/2-1, SE1885/2-2, and SE1885/4-1 and the Deutsche Krebshilfe through grant 70112628.

## Conflict of Interest

The authors declare that the research was conducted in the absence of any commercial or financial relationships that could be construed as a potential conflict of interest.

## Publisher’s Note

All claims expressed in this article are solely those of the authors and do not necessarily represent those of their affiliated organizations, or those of the publisher, the editors and the reviewers. Any product that may be evaluated in this article, or claim that may be made by its manufacturer, is not guaranteed or endorsed by the publisher.

## Supplementary Material

Supplementary Figure 1 Impact of age on the sMZ B cell heterogeneity of five healthy donors. **(A)** Unsupervised t-SNE analysis of most differentially expressed genes (MANOVA q < 0.05, 31 genes, **Table S2**) among sMZ B cells from five donors. The five donors are separately displayed. **(B)** Supervised PCA of 23 genes associated with hallmark molecules distinguishing NBCs from MBCs, and sMZ B cells, and NOTCH-signalling genes (**Table S2**). The five donors are separately displayed.
