# Supplementary figures and images for: The Splenic Marginal Zone in Children Is Characterized by a Subpopulation of CD27-Negative, Lowly IGHV-Mutated B Cells
Source: Front Immunol. 2022 Jan 27;13:825619. doi: 10.3389/fimmu.2022.825619 (PMC8828478; doi:10.3389/fimmu.2022.825619)

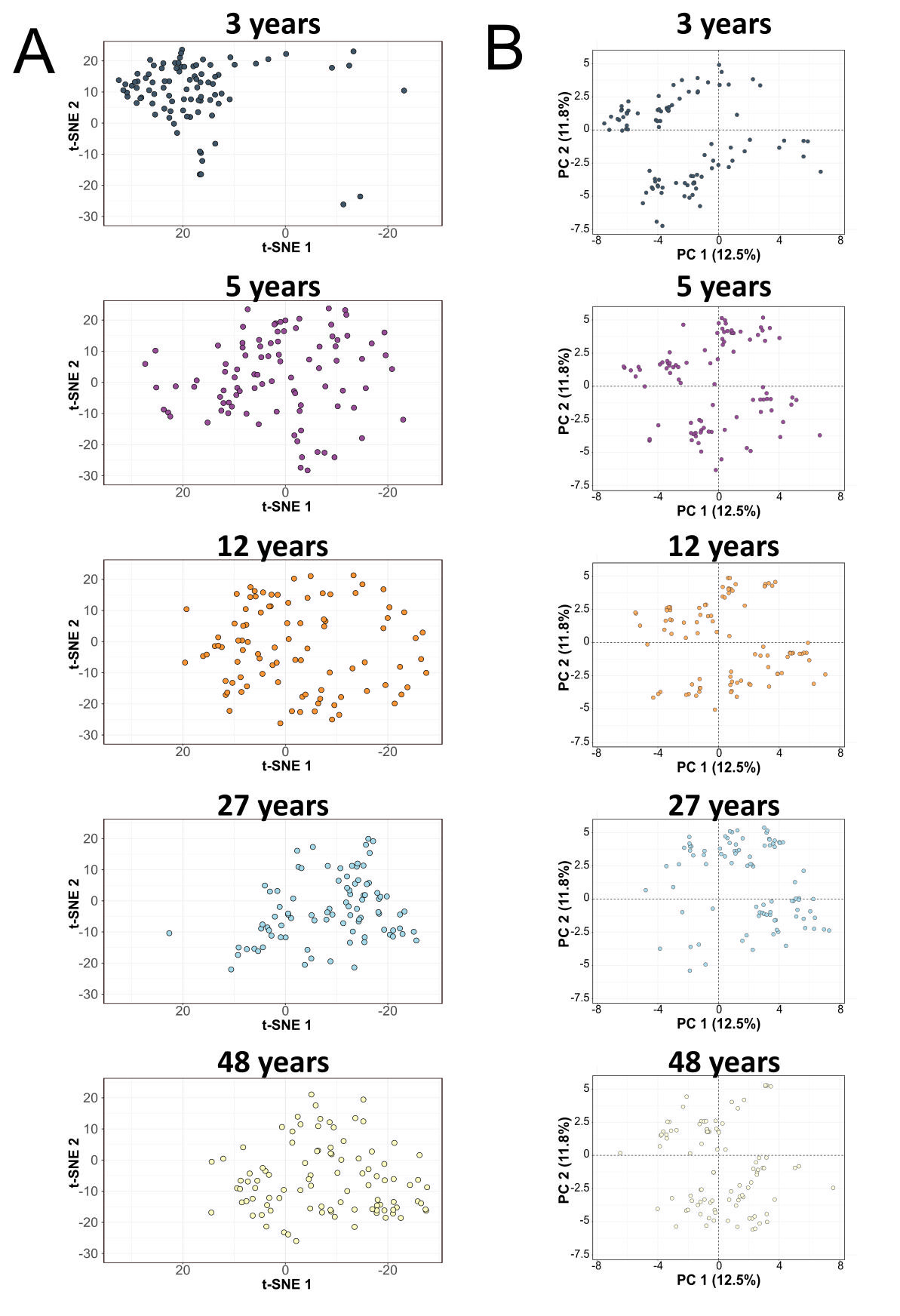

Supplement: Supplementary Figure 1 — Impact of age on the sMZ B cell heterogeneity of five healthy donors. (A) Unsupervised t-SNE analysis of most differentially expressed genes (MANOVA q < 0.05, 31 genes, Table S2 ) among sMZ B cells from five donors. The five donors are separately displayed. (B) Supervised PCA of 23 genes associated with hallmark molecules distinguishing NBCs from MBCs, and sMZ B cells, and NOTCH-signalling genes ( Table S2 ). The five donors are separately displayed. [file Image_1.tif]
